# Some Practical Notes

**Published:** 1889-06

**Authors:** H. C. Morrow


### SOME PRACTICAL NOTES.

I received the first installment of the Repertory, and am delighted with it. I think every true homœopath should have this Repertory. Since you invite criticism, I am sorry you did not put down the rubric "Visions on closing the eyes." It is true you have this heading under "Faces" and "Spectres," but "Visions" embraces all, Faces, Images, Spectres, etc. The combined list of remedies under these two headings is not complete, if you mean *all* the remedies that have "Visions on closing the eyes." When you arrive at "Rectum and Stool" under the rubric, "Odor like rotten eggs," I hope it will be more complete than Bell's last edition, "Diarrhœa, etc." Under that rubric he omits Sulphur.

I cured four cases of diarrhœa (Sulph.$^{cm}$ Fincke) a year ago where this was the principal and almost only odor. I

was raised on a farm, and have had rotten eggs to burst in my face, and I know what the odor is. One of the above-mentioned cases was of many years standing. I consider it (this symptom) almost as characteristic of Sulphur as of Chamomilla, and should be printed in italics. My list complete under this rubric is: Arsen. alb., Asclep., *Calc-c.*, Carbol. ac., Carlsbad, CHAM., Fagop., Hep-s., *Psor.*, Spruedel, Staph., *Sulph.*, Sulph. ac., Wiesbaden.

I am sorry you did not include Sanicula in your Repertory. I consider it the most valuable addition to our materia medica of recent years. It takes a place alongside of Calc-c., Lycop., and Sulphur. It is of great value in the treatment of the diarrhœas and summer complaints of children. I cured last fall with the 50M (F.) a little baby of diarrhœa which had been sick the whole summer under the care of a mongrel. If I may so call it, Sanicula is the chronic of Chamomilla. A peculiar clinical symptom in the above case was that *he wanted to lie on something hard*, although as poor as a snake. I have been suffering since I was a lad from the effects of suppressed itch. I stated my case to Dr. Sherbino, who gave me Sanicula, and I am in better health now than for twenty years. Good prescribers had before failed to give me any lasting relief. I had the symptom, "felt as if he had on cold, damp stockings," but Calcarea would not touch it. Also "Sweat about the head and neck when asleep, wetting the pillow far around."

I have a case of chills, which has the symptom, during chill, "feels all over body as if packed in wet salt." Also chilliness (flashes of), starting from spine in lumbar region, and instantaneously like lightning, running up back to top of head, down the legs, and to the feet (same case).

I also recently had a case of gall-stone colic, with the following symptom: During the severest paroxysms of pain wanted to sit up (erect) and *pull* against something with all her might. Can you give me the remedies which cover them? If not, will you publish in the PHYSICIAN? I hope I have not imposed on you. H. C. MORROW.

